# Neurogenic Detrusor Overactivity Is Associated with Decreased Expression and Function of the Large Conductance Voltage- and Ca^2+^-Activated K^+^ Channels

**DOI:** 10.1371/journal.pone.0068052

**Published:** 2013-07-05

**Authors:** Kiril L. Hristov, Serge A. Y. Afeli, Shankar P. Parajuli, Qiuping Cheng, Eric S. Rovner, Georgi V. Petkov

**Affiliations:** 1 Department of Drug Discovery and Biomedical Sciences, South Carolina College of Pharmacy, University of South Carolina, Columbia, South Carolina, United States of America; 2 Medical University of South Carolina, Charleston, South Carolina, United States of America; Dalhousie University, Canada

## Abstract

Patients suffering from a variety of neurological diseases such as spinal cord injury, Parkinson’s disease, and multiple sclerosis often develop neurogenic detrusor overactivity (NDO), which currently lacks a universally effective therapy. Here, we tested the hypothesis that NDO is associated with changes in detrusor smooth muscle (DSM) large conductance Ca^2+^-activated K^+^ (BK) channel expression and function. DSM tissue samples from 33 patients were obtained during open bladder surgeries. NDO patients were clinically characterized preoperatively with pressure-flow urodynamics demonstrating detrusor overactivity, in the setting of a clinically relevant neurological condition. Control patients did not have overactive bladder and did not have a clinically relevant neurological disease. We conducted quantitative polymerase chain reactions (qPCR), perforated patch-clamp electrophysiology on freshly-isolated DSM cells, and functional studies on DSM contractility. qPCR experiments revealed that DSM samples from NDO patients showed decreased BK channel mRNA expression in comparison to controls. Patch-clamp experiments demonstrated reduced whole cell and transient BK currents (TBKCs) in freshly-isolated DSM cells from NDO patients. Functional studies on DSM contractility showed that spontaneous phasic contractions had a decreased sensitivity to iberiotoxin, a selective BK channel inhibitor, in DSM strips isolated from NDO patients. These results reveal the novel finding that NDO is associated with decreased DSM BK channel expression and function leading to increased DSM excitability and contractility. BK channel openers or BK channel gene transfer could be an alternative strategy to control NDO. Future clinical trials are needed to evaluate the value of BK channel opening drugs or gene therapies for NDO treatment and to identify any possible adverse effects.

## Introduction

Overactive bladder (OAB) is described as urgency, with or without incontinence, usually associated with frequency and nocturia [1]. Patients with various neurological diseases often develop voiding dysfunction which presents clinically as OAB [2]. These OAB symptoms are often caused by dysfunction of the neurological control mechanisms subserving bladder function. When such a condition is the result of urodynamically demonstrable involuntary bladder contractions, it is termed neurogenic detrusor overactivity (NDO). The pathology of NDO is often associated with alteration of the electromechanical properties of the detrusor smooth muscle (DSM), including increased DSM excitability [2]. Aside from the clinical symptoms of frequency, urgency and incontinence, high pressure involuntary contractions of DSM in patients with NDO may eventually lead to irreversible changes in DSM. Such changes may result in decreased bladder compliance with associated high intravesical pressure during the bladder filling phase, and if left untreated may lead to deterioration of the upper urinary tract [3]–[5].

Currently, there is not an optimal pharmacological agent to treat NDO [6]. Antimuscarinics are used to treat NDO but these agents have limited effectiveness and, due to a lack of specificity for the lower urinary tract, are associated with collateral undesirable adverse effects elsewhere in the body [7]–[12]. The selective β3-adrenoceptor agonist mirabegron [13], [14] has been recently proposed to treat OAB, however its effectiveness in patients with NDO remains uncertain. Newer therapies such as intravesical botulinum toxin [3], [15] are not only invasive and expensive but are also associated with safety concerns [3], [6], [16]. Therefore, novel approaches to treat NDO are urgently needed. A critical step for the development of a new, safe, and more effective therapy for NDO is developing a better understanding of the etiology of NDO and the basic mechanisms that control DSM excitability and contractility in NDO patients.

NDO is characterized by increased spontaneous phasic DSM contractions during the filling phase of urodynamics in an individual with a clinically relevant neurological condition [17], [18]. The underlying basis of these spontaneous phasic DSM contractions is the spontaneous action potentials [19]. A number of different types of K^+^ channels control DSM action potential generation [20]. The large conductance voltage- and Ca^2+^-activated K^+^ (BK) channel is arguably the most important physiologically relevant K^+^ channel involved in the regulation of the DSM action potential, the resting membrane potential, and DSM contractility [20]–[32]. Iberiotoxin, a selective blocker of the BK channel, inhibits the majority of the whole cell outward K^+^ current, depolarizes the DSM cell resting membrane potential, and increases the contractility of human isolated DSM strips [29]. Because of their prominent physiological role in DSM excitability and contractility, BK channels have been identified as a valid target for the pharmacological or genetic control of OAB [18], [21], [27], [29], [31], [33]–[37].

The absence of pore-forming BKα subunits or regulatory BKβ1 subunits significantly increases DSM contractility and urination frequency in association with detrusor overactivity (DO) [24], [26], [30], [38]. In a rat model of partial urethral obstruction, there was a significant decrease in whole cell BK channel current associated with over a 2-fold reduction in BKα subunit mRNA and protein expression [39]. Recent studies also demonstrated direct involvement of BK channels in the etiology of OAB in patients with benign prostatic hyperplasia (BPH) and DO [33] as well as NDO [18]. These results reinforce the notion of a significant role for the BK channel in DSM function and dysfunction, and suggest that BK channel dysfunction can lead to the OAB phenotype. However, the role of the BK channel in the pathophysiology of NDO has not been investigated.

Here, we used a multidisciplinary experimental approach utilizing qPCR and patch-clamp electrophysiology on freshly-isolated human DSM cells, functional studies on human DSM tissue strip contractility, as well as pharmacological protocols to evaluate if NDO is associated with changes in the DSM BK channel expression and function.

## Materials and Methods

### Human DSM Tissue Collection

All procedures for human DSM tissue collection were reviewed and approved by the Institutional Review Board of the Medical University of South Carolina (MUSC) under the protocol HR 16918. According to this protocol, human DSM tissues were collected upon written informed consent to participate in this study. Human DSM tissue samples from control and NDO patients were obtained during open bladder surgeries performed for a variety of indications such as bladder cancer, urothelial carcinoma, and ureteral reimplantation for repair of urinary fistula. NDO patients were clinically characterized preoperatively with pressure-flow urodynamics demonstrating DO in the setting of a clinically relevant neurological condition such as spina bifida, cerebral palsy, and spinal cord injury; with or without an American Urological Association (AUA) symptom score >7. Control patients did not have OAB (defined as an AUA symptom score <8) and did not have a clinically relevant neurological disease. In this study, 33 patients (19 males and 14 females; 26 Caucasians and 7 African-Americans; 62.4±2.8 years average age) were used. From those 33 patients, 4 were with clinically confirmed NDO (4 females; 2 Caucasians and 2 African-Americans; 46.5±14.8 years average age), and 29 patients (19 males and 10 females; 24 Caucasians and 5 African-Americans; 64.6±2.4 years average age) were without NDO and were used as controls. The DSM specimens were obtained from tumor free regions of the bladder dome, posterior or anterior bladder wall. Two types of DSM samples were collected from each patient. The first sample was stored in ice-cold Ca^2+^-free *N*-2-hydroxyethylpiperazine-*N*'-2-ethanesulphonic acid (HEPES)-buffered dissection solution (**§Solutions and Drugs**) and was used to conduct patch-clamp and functional studies on DSM contractility. The second sample was kept in RNAlater solution (QIAGEN GmbH, Hilden Germany) and was used for qPCR experiments. Both samples were transported under controlled conditions to the laboratory after surgery.

### DSM Cell Isolation for Patch-clamp Experiments

Human DSM cells for patch-clamp electrophysiological experiments were enzymatically isolated as previously described [21], [29], [40].

### Quantification of BK Channel Subunits mRNA Message by qPCR

Total RNA was extracted from both control and NDO DSM tissue using the RNeasy Mini Kit (Qiagen, Hilden, Germany), then reverse-transcribed into cDNA using oligo d(T) primers (Promega, WI, USA), M-MLV Reverse Transcriptase (Promega, WI, USA), dNTP mix (Fermentas Life Sciences, ME, USA) and RNase inhibitor (Applied Biosystems, USA) as previously described [21], [29], [40]. Specific primers for BKα, BKβ1, BKβ4 subunits, and GAPDH were designed based on the cDNA complete sequences of human genes in Genbank and synthesized by Integrated DNA Technologies (IDT, Coralville, Iowa, USA) (**Table 1**). qPCR experiments followed a two-step amplification plus melting curve protocol using the IQ^TM^5 Thermo Cycler system (Bio-Rad, California, USA). qPCR experiments were carried out on human DSM tissue cDNA (0.5 µg/µL) using IQ SYBR Green Supermix (Bio-Rad, California, USA). GAPDH was chosen as an internal control gene to analyze BKα, BKβ1, and BKβ4 subunits mRNA relative expression and each sample was run in triplicate. The parameters of the qPCR experiments were as follows: Cycle 1, 95°C for 3 min; cycle 2, 95°C for 10 s, then 61°C for 30 s (repeated 40 times). The melting curve analysis was then run between 61°C and 101°C with 0.5°C increments every 10 s. qPCR products were purified using the GenElute™ PCR Clean-Up Kit (Sigma-Aldrich Co., St. Louis, MO, USA) and sequenced at the University of South Carolina Environmental Genomics Core Facility to confirm their identity.

**Table 1:** qPCR primers for BKα, BKβ1, BKβ4 subunits and GAPDH.

	Sense	Anti-sense	Product (bp)	Accession Number
BKα	TGCCTAAAGCATGATTTG	GCCGACATGCTAAATAAATTAG	400	NG012270
BKβ1	TGCCACCTGATTGAGACC	TGCGGAGAAGCAGTAGAAG	258	NM004137
BKβ4	CATTTGTGGTGGGCTTTCT	ACATGTTCCGCAGGTGG	168	NM170782
GAPDH	GGATTTGGTCGTATTGGG	GGAAGATGGTGATGGGATT	205	NM002046

bp – base pairs.

### Electrophysiological (Patch-clamp) Recordings

The amphotericin-B perforated whole cell configuration of the patch-clamp technique was used [41], [42]. This approach allows the preservation of the native physiological environment of human DSM cells. Whole cell currents were recorded using an Axopatch 200B amplifier, Digidata 1440A, and pCLAMP version 10.2 software (Molecular Devices, Union City, CA). The signals were filtered by an eight-pole Bessel filter 900CT/9L8L (Frequency Devices). The patch-clamp pipettes were made from borosilicate glass (Sutter Instruments, Novato, CA), coated with dental wax to reduce capacitance, and polished with a Micro Forge MF-830 fire polisher (Narishige Group, Tokyo, Japan) to give a final tip resistance of 4–6 MΩ. All electrophysiological experiments were conducted at room temperature (22–23°C).

### Isometric DSM Tension Recordings

5–7 mm long and 1–2 mm wide mucosa-free DSM strips were excised from human DSM tissue for functional studies as previously described [21], [29], [40]. Briefly, human DSM isolated strips were secured between clips and mounted between a force displacement transducer and a stationary hook. The strips were then submerged in thermostatically controlled tissue baths with Ca^2+^-containing physiological saline solution (PSS) at 37°C and aerated with 95% O_2_/5% CO_2_. Next, the DSM strips were stretched to approximately 1.0 g force and were allowed to equilibrate for 1 h. During this equilibration period, strips were washed out with fresh PSS every 15 min.

### Solutions and Drugs

The Ca^2+^-free dissection solution had the following composition (in mM): 80 monosodium glutamate, 55 NaCl, 6 KCl, 10 glucose, 10 HEPES, 2 MgCl_2_, and pH 7.3 adjusted with NaOH. The Ca^2+^-containing PSS used in DSM tension recording experiments was prepared daily and contained (in mM): 119 NaCl, 4.7 KCl, 24 NaHCO_3_, 1.2 KH_2_PO_4_, 2.5 CaCl_2_, 1.2 MgSO_4_ and 11 glucose, and aerated with 95% O_2_/5% CO_2_ to obtain pH 7.4. The extracellular (bath) solution used in the patch-clamp experiments had the following composition (in mM): 134 NaCl, 6 KCl, 1 MgCl_2_, 2 CaCl_2_, 10 glucose, and 10 HEPES, pH adjusted to 7.4 with NaOH. The pipette solution used in the patch-clamp experiments contained (in mM): 110 potassium aspartate, 30 KCl, 10 NaCl, 1 MgCl_2_, 10 HEPES, and 0.05 EGTA, pH adjusted to 7.2 with NaOH and supplemented with freshly dissolved (every 1–2 h) 200 µg/ml amphotericin-B. Iberiotoxin and tetrodotoxin (TTX) were purchased from Sigma-Aldrich Co.

### Data Analysis and Statistics

mRNA relative expression was analyzed using the comparative threshold (Ct) method (ΔΔCt, delta-delta Ct) [43] after determining the Ct values for reference (GAPDH) and target genes (BKα, BKβ1, and BKβ4 subunits) for each sample. Fold-changes in target mRNA expression level were calculated after normalization to GAPDH. MiniAnalysis (Synaptosoft, Inc., Decatur, GA) software version 6.0.7 was used to analyze data and GraphPad Prism 4.03 software (GraphPad Software Inc., San Diego, CA) was used for further statistical analysis. To compare the phasic contraction parameters, data were normalized to the control spontaneous contractions (100%) prior to addition of iberiotoxin and expressed as percentages. Clampfit 10.2 software (Molecular Devices, Inc.) was used for the data analysis of the patch-clamp experiments. The data and original recordings were illustrated using GraphPad Prism and CorelDraw Graphic Suite X3 (Corel Co., Ottawa, Canada) software. Results are summarized as mean±SE where **N** represents the number of patients, **n** represents the number of human DSM isolated strips, PCR samples, or human DSM cells. Data were compared using two-tailed paired or unpaired Student’s t-test. A P-value <0.05 was considered statistically significant.

## Results

### The Relative Expression Levels of BKα Subunit mRNA is Significantly Decreased in NDO DSM

**Figure 1:**
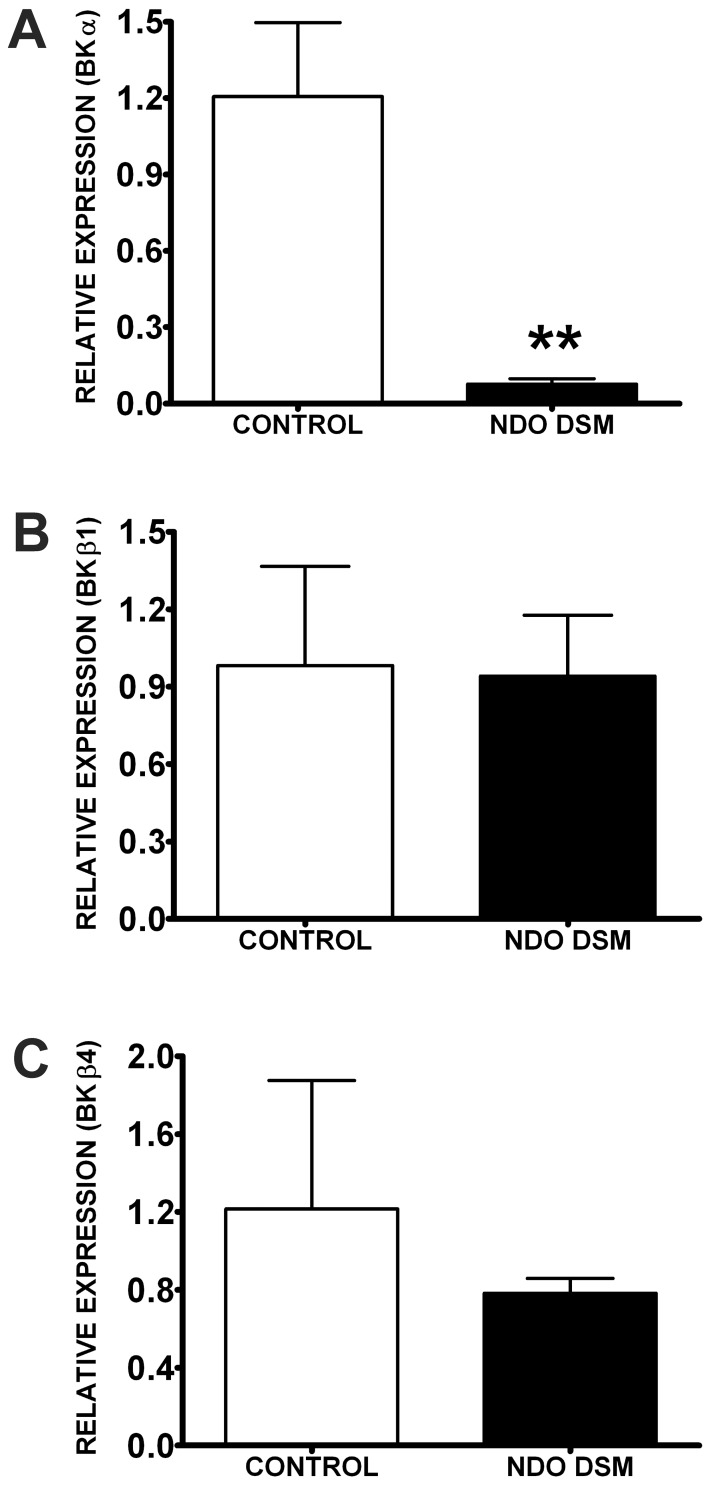
Patients with NDO have decreased BKα subunit mRNA expression in DSM. qPCR analyses showing 15.8-fold, 1.1-fold, and 1.6-fold decreases in BKα (**A**), BKβ1 (**B**), and BKβ4 subunits (**C**) mRNA expression in NDO DSM tissue (n = 3, N = 3) compared to controls (n = 7, N = 7; **P<0.01). Data are shown as relative mRNA expression normalized to GAPDH.

Recently, we established that human DSM cells express BKα, BKβ1, and BKβ4 subunits at both mRNA and protein levels [29]. Here, we revealed that in patients with NDO, the BKα, BKβ1, and BKβ4 subunit mRNA expression was lower compared to control patients (Fig. 1). There was a 15.8-fold decrease in BKα subunit mRNA relative expression in NDO DSM (n = 3, N = 3) compared to controls (n = 7, N = 7; P<0.01; Fig. 1). The changes in BKβ1 and BKβ4 subunits in NDO DSM compared to controls were not statistically significant and were evaluated at 1.1-fold and 1.6-fold decrease, respectively (P>0.05; Fig. 1). Glyceraldehyde-3-phosphate dehydrogenase (GAPDH) was used as an internal control and did not vary significantly between DSM samples from control and NDO patients. These data suggest that in patients suffering from NDO, there is a statistically significant decrease in BKα subunit expression but not in BKβ1 and BKβ4 subunit expression.

### Iberiotoxin-sensitive Whole Cell BK Current is Decreased in NDO DSM Cells

**Figure 2:**
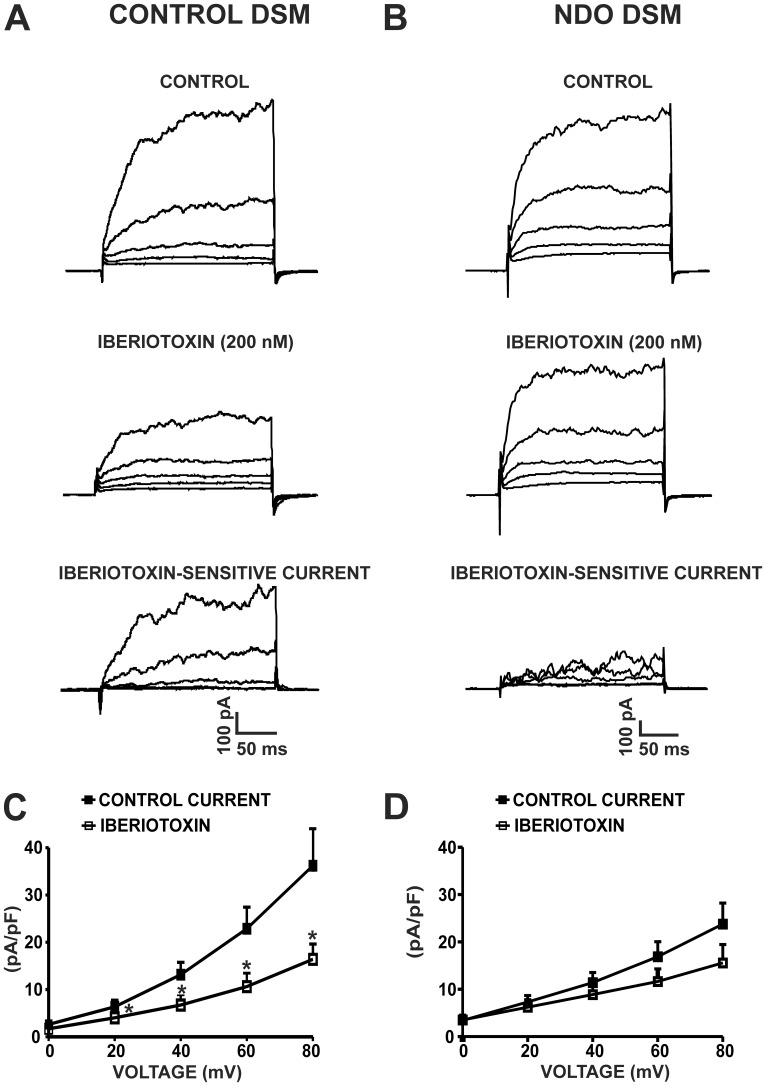
Iberiotoxin-sensitive steady-state BK current is significantly reduced in human NDO DSM cells compared to controls. Original recordings illustrating the effect of 200 nM iberiotoxin on the voltage-dependent steady-state whole cell current in a control DSM cell (**A**) and in an NDO DSM cell (**B**). Current-voltage relationships illustrating the differences in whole cell current density in the absence or presence of 200 nM iberiotoxin in control DSM cells (n = 9, N = 9; *P<0.05) (**C**) and NDO DSM cells (n = 8, N = 4; P>0.05) (**D**).

We recently showed that the BK channel is the major regulator of human DSM excitability [21], [29]. Here, we examined whether DSM BK channel activity changes during NDO. The average human DSM cell capacitance was 24.4±2.7 pF in NDO DSM cells (n = 16, N = 4), and 20.6±2.2 pF in control DSM cells (n = 20, N = 13; P>0.05). Human DSM cells were held at −70 mV and then stepped from 0 mV to +80 mV in 20 mV increments with 200 ms duration. The cells responded with a gradual increase of the whole cell current with each depolarization step (**Fig. 2**). Application of the selective BK channel inhibitor, iberiotoxin (200 nM), significantly decreased the whole cell current in DSM cells from control patients (n = 9, N = 9; P<0.05; **Fig. 2A**). **Figure 2** illustrates that the iberiotoxin-sensitive BK current represented more than 50% of the total outward current in DSM cells from control patients. However, iberiotoxin (200 nM) did not significantly affect the amplitude of the whole cell outward current in DSM cells from NDO patients (**Fig. 2B**). **Figure 2** also illustrates that the iberiotoxin-sensitive BK current represented a very small portion of the total whole cell current in NDO DSM cells, whereas iberiotoxin (200 nM) significantly reduced the whole cell outward current in DSM cells from control patients (**Fig. 2C**). There was no significant difference in the current density before and after application of 200 nM iberiotoxin in NDO DSM cells (n = 8, N = 4; P>0.05; **Fig. 2D**). These data showed that the iberiotoxin-sensitive BK current was significantly reduced in NDO DSM cells.

### Transient BK Currents (TBKCs) Amplitude and Frequency are Decreased in NDO DSM Cells

**Figure 3:**
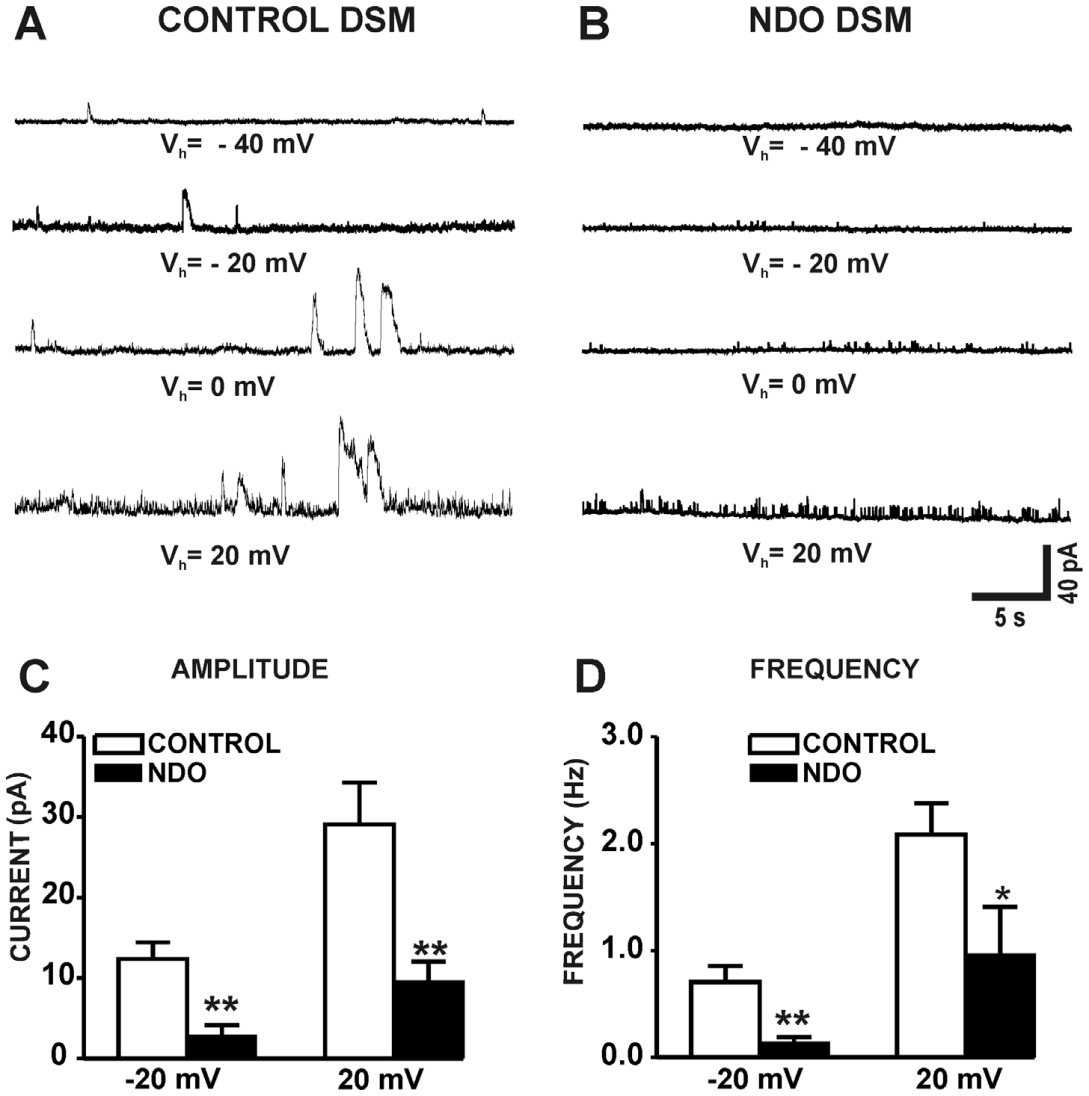
The amplitude and frequency of the transient BK currents (TBKCs) are decreased in NDO DSM cells compared to controls. Original recordings of TBKCs in a control DSM cell (**A**) and an NDO DSM cell (**B**) recorded at different holding potentials. Summary data showing that TBKCs’s amplitude (**C**) and frequency (**D**) were significantly decreased in NDO DSM cells (n = 9, N = 3), compared to control DSM cells (n = 12, N = 10; *P<0.05, **P<0.01).

Recently, we provided the first electrophysiological evidence for the presence of TBKCs in human DSM cells [29]. Here, we examined if the activity of TBKCs in DSM cells changes under pathological conditions of NDO. TBKCs’ amplitude and frequency were significantly reduced in DSM cells in patients with NDO (n = 9, N = 3) compared to the control group (n = 12, N = 10; P<0.05; **Fig. 3**). The results indicate that TBKC activity was significantly reduced in NDO DSM cells.

### Pharmacological Inhibition of the BK Channels with Iberiotoxin has a Reduced Effect on the Contractility of DSM Strips Isolated from Patients with NDO

**Figure 4:**
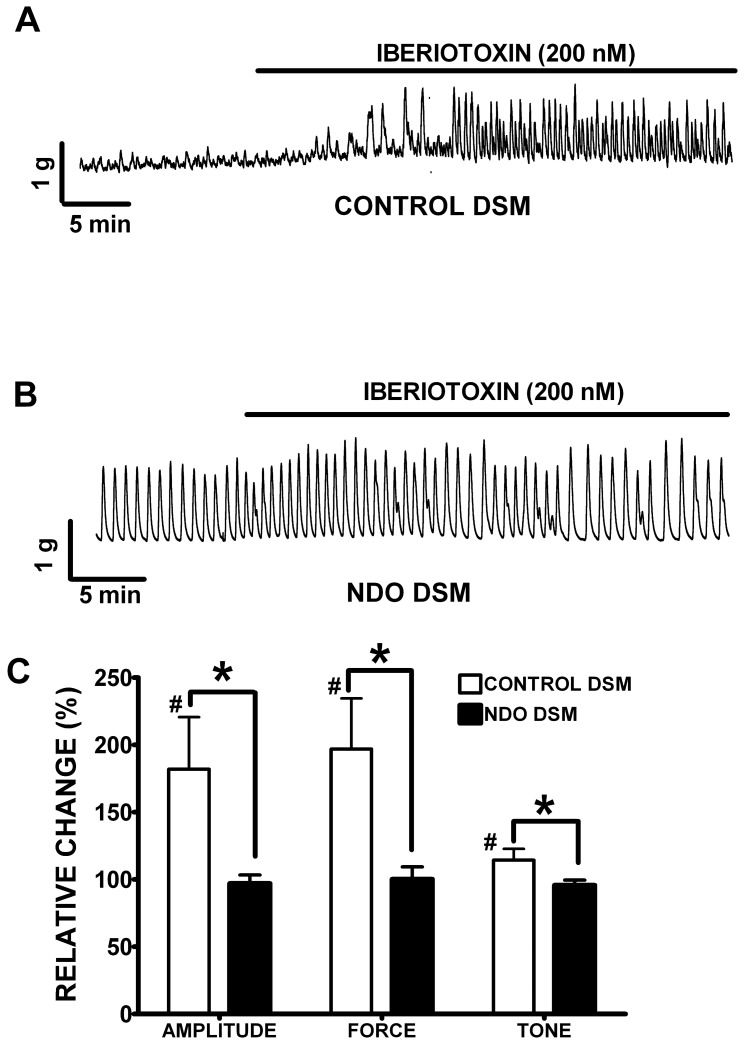
Inhibition of the BK channel with iberiotoxin causes a significant increase in spontaneous phasic and tonic contractions in control DSM but not in NDO DSM isolated strips. Original DSM tension recordings illustrating iberiotoxin (200 nM) effects on spontaneous phasic contractions in control (**A**) and NDO (**B**) DSM isolated strips. (**C**) Summary data showing a statistically significant increase in spontaneous phasic contraction amplitude, muscle force integral, and muscle tone of control DSM strips (n = 12, N = 7; P<0.05), and a lack of significant iberiotoxin effects in NDO DSM strips (n = 13, N = 7; P>0.05). #P<0.05 for control vs. iberiotoxin and *P<0.05 for control vs. NDO.

To restrain neuronal activity, TTX (1 µM), a selective voltage-gated Na^+^ channel blocker, was added to the bath in the beginning of all experiments. In DSM strips isolated from control patients, iberiotoxin (200 nM) considerably increased the phasic contraction amplitude, muscle force integral, and muscle tone by 81.9±38.7%, 96.9±37.7%, and 14.4±8.3%, respectively (n = 12, N = 7; P<0.05; **Fig. 4A, C**). However, in DSM strips isolated from patients with NDO, iberiotoxin (200 nM) had no significant effects on any of the parameters of DSM contractility, including phasic contraction amplitude, muscle force integral, and muscle tone (n = 13, N = 3; P>0.05; **Fig. 4B, C**). Collectively, these results suggest a decrease in BK channel activity in DSM from patients with NDO, which contributes to the increase in the spontaneous phasic contractions.

## Discussion

In the present study, we found significant physiological differences between control and NDO human tissues and cells that reveal important insights into the etiology of NDO. Using a combined multidisciplinary approach, we provide evidence for a critical role of the BK channel in the pathophysiology of NDO and demonstrate a significant decrease in the function of the BK channels in the DSM of such patients. Based on our experimental data, we have noted three important key results. First, mRNA expression of the pore-forming BKα subunit was significantly decreased in NDO DSM compared to controls. Secondly, whole cell BK current and TBKCs were both significantly decreased in NDO DSM cells compared to controls. Thirdly, inhibition of the BK channels with iberiotoxin did not significantly affect the spontaneous NDO DSM contractions, but did so in control DSM tissues, supporting the concept that NDO tissue is fundamentally biologically altered.

Recently, we provided molecular evidence for the expression of the BKα, BKβ1, and BKβ4 subunits in human DSM cells at both the mRNA and protein levels [29]. Here, we investigated whether the expression of BK channel subunits was altered in DSM from NDO patients. The results showed a 15.8-fold decrease of mRNA expression of the pore-forming BKα subunit but no statistical changes in BKβ1 and BKβ4 subunit mRNA expression in NDO DSM as compared to control DSM (**Fig. 1**). This is the first molecular evidence for decreased BK channel expression in DSM from NDO patients. The BKα subunit plays a critical role in DSM function, since it forms the functional pore of the BK channel [24], [28], [30], whereas the DSM BKβ1 subunits have a regulatory role by increasing BK channel Ca^2+^ sensitivity and modifying BK current activity [20], [26]. The functional role of BKβ4 subunit in DSM is still unclear [29]. Therefore, any changes in the BKα or BKβ1 subunits’ molecular expression could significantly impact DSM excitability and contractility. This has been well demonstrated in studies with genetically modified mice [24], [26], [28], [30], [38]. Deletion of the BKα subunit depolarizes DSM cell membrane potential [24], [28], increases DSM contractility [24], [30], and results in a phenotype consistent with DO [24], [30], [38]. The absence of the smooth muscle specific BKβ1 subunit causes an increase in DSM contractility in mice, which suggests that BKβ1 subunit is also an important regulator of DSM function [26]. Decreased mRNA expression of BKα and BKβ1 subunits was observed in animal models of partial urethral outlet obstruction [33], [39], [44]. Furthermore, patients with BPH and associated DO had a significantly reduced mRNA and protein expression of BKα and BKβ subunits [33]. Our results are consistent with the above study, as we demonstrated that in NDO patients, the mRNA expression of BKα subunit is significantly decreased. These studies support the concept that the NDO phenotype is associated with decreased BK channel expression in DSM.

Recently, we demonstrated that in DSM cells from patients with no history of OAB symptoms, the BK channels carry the majority of the steady-state whole cell current and are responsible for the generation of TBKCs [21], [29]. Here, we sought to investigate the role of BK channels in NDO. BK channel function has not previously been investigated in NDO DSM using the perforated patch-clamp technique, which is the only method that can directly access ion channel activity while preserving intracellular signaling mechanisms. Our patch-clamp data showed that the iberiotoxin-sensitive BK current was significantly reduced in DSM cells from NDO patients compared to control patients. Furthermore, TBKCs’s amplitude and frequency were significantly decreased in DSM cells from patients with NDO compared to controls (**Fig. 3**). The patch-clamp results are consistent with our qPCR data, and illustrate that BK channel function is significantly reduced in NDO DSM compared to control DSM.

In patients without symptoms of OAB, the BK channel is also a major regulator of DSM contractility [29]. In the present study, we investigated how the decreased activity of the BK channel affects DSM phasic contractions in NDO patients. Unlike control DSM, inhibition of the BK channel with iberiotoxin did not significantly affect any of the parameters of spontaneous DSM contractility in NDO DSM isolated strips (**Fig. 4**). These results are supported by a recent study reporting no effect of iberiotoxin or NS1619, a BK channel opener on DSM phasic contractions in patients with NDO [18]. The results from our functional studies support the concept that BK channel activity is decreased in NDO DSM.

The complex etiology of NDO is not completely understood. Our results provide strong evidence that decreased BK channel activity is associated with NDO. Teleologically, this may be a consequence of a compensatory or adaptive mechanism in neurologically affected individuals that leads to increased DSM contractility allowing optimization of bladder emptying in the absence of normal innervation. Similar decreases in BK channel expression and activity have been reported in DSM from patients with BPH and DO, which is a condition with a different etiology than NDO [33]. One of the possible explanations for these similarities is that both conditions, regardless of their etiology, utilize similar compensatory mechanisms to decrease the expression of BK channels in DSM, and thus to alter DSM contractility.

Recently, we found that the selective BK channel opener NS1619 significantly decreased human DSM excitability and contractility [21]. We expect that a selective BK channel opener would be a highly effective treatment for NDO. However, currently available agents are not selective for DSM tissue resulting in collateral effects elsewhere in the body. Nevertheless, there is an ongoing effort to develop a new class of more potent and selective BK channel openers [45]. A genetic approach to enhance BK channel expression could also be a successful therapeutic strategy. Injection of BK channel naked DNA has been shown to eliminate bladder overactivity caused by partial urethral outlet obstruction in rats [35]. A clinical Phase I safety trial of plasmid based gene therapy using BK channel *hSlo cDNA* has been completed for erectile dysfunction and a future clinical trial is planned for the treatment of patients with OAB [36], [37].

In conclusion, this study provides a significant contribution to our basic understanding of the key functional role of the BK channels in NDO. We demonstrated a decrease in the mRNA expression level of the pore-forming BKα subunit in DSM from NDO patients. Furthermore, we provided evidence for BK channel activity in NDO DSM by using the perforated patch-clamp approach, and revealed decreased whole cell BK currents and TBKCs in the DSM cells from NDO patients. These results are consistent with our functional studies showing a lack of an effect of iberiotoxin on spontaneous contractility in NDO DSM isolated strips. These findings support the pursuit and investigation of novel treatments for NDO which target the BK channel. Our results support the concept that BK channel openers and BK channel gene transfer could be an alternative strategy to control NDO as compared to currently prescribed antimuscarinics.
